# Imaging the lifecycle of *Microsporidia* sp. MB in *Anopheles coluzzii* from western Burkina Faso reveals octosporogony

**DOI:** 10.1128/msphere.00851-24

**Published:** 2025-05-22

**Authors:** Ewan R. S. Parry, Roland Pevsner, Beth C. Poulton, Deepak-Kumar Purusothaman, Abdelhakeem I. Adam, Sare Issiaka, Thomas H. Ant, Stephanie M. Rainey, Etienne Bilgo, Abdoulaye Diabaté, Steven P. Sinkins

**Affiliations:** 1 School of Infection and Immunity, University of Glasgow and MRC-University of Glasgow Centre for Virus Research, Glasgow, United Kingdom; 2 Institut de Recherche en Sciences de la Santé (IRSS), BoboDioulasso, Burkina Faso; University of Michigan Medical School, Ann Arbor, Michigan, USA

**Keywords:** endosymbionts, malaria, *Anopheles*, microsporidia, mosquito

## Abstract

A newly discovered microsporidian, *Microsporidia* sp. MB (MB), was recently found to block *Plasmodium falciparum* transmission in *Anopheles arabiensis* mosquitoes from Kenya. Here, we describe the lifecycle of the first laboratory colony of *Anopheles coluzzii* with MB, originating from western Burkina Faso. The lifecycle of MB was explored using fluorescent *in situ* hybridization and confocal microscopy, facilitated by the development of optimized protocols to produce histological sections of whole adult, larval, and embryo tissues. As in *An. arabiensis*, transmission appears to be predominantly vertical, with MB highly localized to the ovaries across multiple lifecycle stages. MB was sparsely distributed within the majority of developing oocytes in the gravid female. After oviposition, in the majority of embryos, MB relocated to the developing gonad at the onset of tissue differentiation, suggesting a highly specialized adaptation to host tissues. Sporogony was identified for the first time in a proportion of developing oocytes and in embryos post-oviposition. Microsporidian spore characteristics were subsequently confirmed with electron microscopy. Identification of MB sporogony in eggs suggests there are alternative horizontal routes of transmission which could play an important role in developing MB as a malaria control strategy.

**IMPORTANCE:**

Malaria in West Africa, caused by *Plasmodium falciparum* infection and spread by anopheline mosquitoes, is responsible for hundreds of thousands of deaths annually and resulted in over 120 million cases in 2022 . The transmission-blocking effect of *Microsporidia* sp. MB (MB) suggests its potential as an agent for combating the spread of malaria. Understanding the routes of transmission and their effect on MB in mosquito populations is crucial for its development as a control tool. The identification of MB spores reveals the potential for another avenue of transmission beyond the vertical transmission from female to offspring. Spores could also have the potential for alternative MB dissemination methods, alongside or instead of adult mosquito releases.

## INTRODUCTION

Malaria continues to be an overwhelming global health challenge, with an estimated 260 million cases and over half a million deaths in 2023 alone (1). In addition to mortalities, the impact of the many more non-fatal cases is severe, reducing job prospects, access to education and contributing to chronic instability and poverty in many countries (2). The African continent bears the brunt of the malaria disease burden with around 94% of global malaria cases and 95% of malaria deaths (1). Malaria is a leading human disease burden, but advances in malaria vector control and antimalarial drugs have mitigated this burden dramatically in many areas (2–4). The most successful vector control strategies deployed so far have been indoor residual spraying and long-lasting insecticidal nets (1, 5). Despite these successes, the spread of insecticide resistance and resurgence of malaria cases in previously cleared areas show there is a need for additional, novel control measures (6–10). Resistance to antimalarial drugs, a major problem in southeast Asia, is now emerging in African countries, including Rwanda, Ethiopia, and Uganda (10–13). Combinations of control strategies are often more effective than when used alone, such as the effect of combining indoor spraying or insecticide-coated bed nets with treatment plans using modern antimalarial drugs (14, 15). There is a need for more mosquito control strategies that can be used synergistically with disease treatments at a large scale and low cost. Naturally occurring symbionts have been used to control other infectious diseases in the field, such as the use of *Wolbachia* to reduce dengue virus transmission in *Aedes* mosquitoes (16–18). To date, few naturally occurring microorganisms have been investigated for controlling disease in *Anopheles* malaria vectors.

Microsporidia are opportunistic, obligate intracellular pathogens with common hosts including higher mammals, fish, insects, crustaceans, and single-celled protists. Most microsporidia are highly host specific and can be either monogenetic or digenetic (19). *Microsporidia* sp. MB (MB) was first identified in *Anopheles arabiensis* mosquitoes collected from western Kenya (20) and has been subsequently detected by PCR in multiple other locations in Kenya (20, 21). Initial challenge experiments have demonstrated that it has the potential for blocking *Plasmodium* transmission in *An. arabiensis* mosquitoes (20, 22). The MB-mediated *Plasmodium* blocking effect in *An. arabiensis* was demonstrated using first-generation field-caught mosquitoes and was not associated with a drop in host fecundity or survival (20). In *An. arabiensis,* MB was found in large quantities in the gonad tissues but also in lower quantities in the gut and carcass (23). Transmission was found to be predominantly vertical, although some horizontal transfer was also reported from positive males to negative females during mating (24, 25). While *Plasmodium* blocking has been linked to other mosquito-infecting microsporidia such as *Vavraia* and *Edhazardia*, these species are highly pathogenic to the adult host (26–31). In West Africa, MB has since been found in *Anopheles coluzzii* in Ghana and Niger, and in *Anopheles gambiae* and *An. coluzzii* in Benin (32–35). In western Burkina Faso, MB was found at medium prevalence in several different sites in *An. coluzzii* and *An. gambiae* mosquitoes. In this study, visualization of MB in different mosquito lifecycle stages was carried out in a newly established lab colony of *An. coluzzii* from Burkina Faso, using fluorescence *in situ* hybridization (FISH) and confocal microscopy.

## RESULTS

### *Microsporidia* sp. MB FISH reveals gonad-specific localization in larvae and pupae

**Fig 1:**
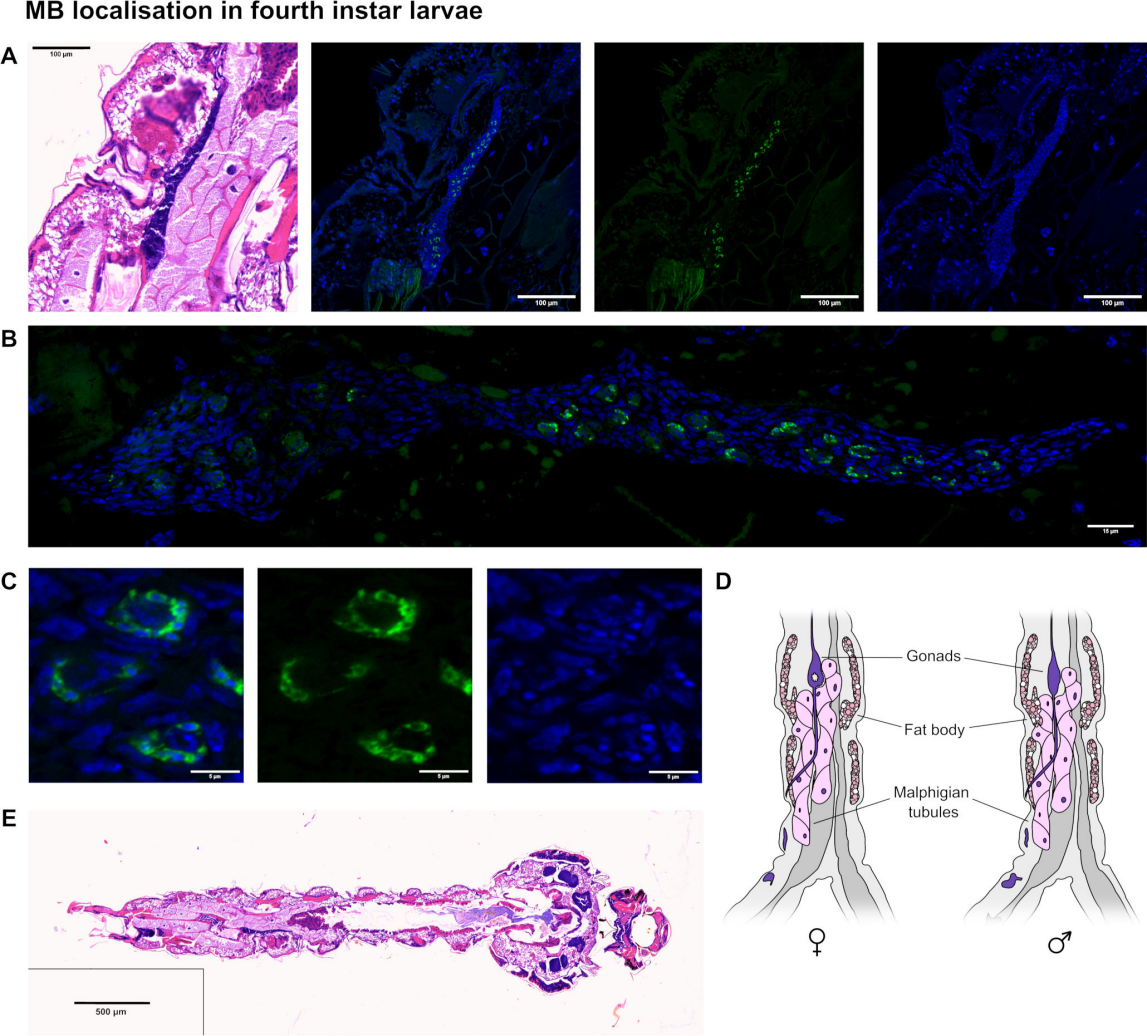
*Microsporidia* sp. MB in histological sections of fourth instar larvae. (**A**) A histological section of a fourth instar larva stained with H&E, showing the developing gonads in dark blue. A proximal section shows MB restricted to this tissue (scale bar =100 µm). MB = green: Alexa Fluor 488-18S FISH probe; nuclei = blue: Hoechst. (**B**) A maximum intensity projection of a multi-tile z-stack acquisition of the whole gonad tissue from the same FISH-stained section described in panel A (scale bar = 15 µm). (**C**) A cropped region of panel B shows MB grouped in spherical clusters, with small individual nuclei compared to larger host nuclei (scale bar =5 µm). (**D**) Diagram showing the localization of gonad tissue in mosquito larval sections (redrawn using references 36, 37). (**E**) The full histological section of the fourth instar larva, including the cropped region from panel A stained in H&E.

To uncover more details of the lifecycle and transmission of MB, different mosquito lifecycle stages of a stable MB-positive lab colony of *An. coluzzii* were arranged and embedded to produce serial formaldehyde-fixed, paraffin-embedded (FFPE) sections. Due to their small size, early larval instars are difficult to section, but by using an optimized mounting protocol, multiple fourth instar larvae can be sectioned at once. Over four experimental replicates, medial sections of multiple fourth instar larvae were either stained with MB-specific Alexa Fluor 488-18S FISH probes and Hoechst (Fig. 1A through C) or with hematoxylin and eosin (H&E) (Fig. 1A and E). No evidence of MB was found in tissues outside of the developing gonad by microscopy. MB was found at high intracellular density within the developing gonad region (Fig. 1A and B); either evenly distributed, or more commonly as spherical clusters around a central host nucleus (*n* = 9) (Fig. 1C). These clusters, seen here as rings in cross-section, were ~5 µm–10 µm in diameter and were distributed evenly throughout the gonad tissue. Gonad tissue can be identified by comparing the diagram of larval posterior characteristics (Fig. 1D) to the full larval section (Fig. 1E). Individual MB nuclei (Hoechst stain) can be differentiated easily from the host nuclei due to their small size. Sections of male and female pupae showed the same MB distribution as in larvae, with spherical clusters of meronts restricted to the gonadal tissues (Fig. S2).

### *Microsporidia* sp. MB localizes to the ovaries in adult female *An. coluzzii*

**Fig 2:**
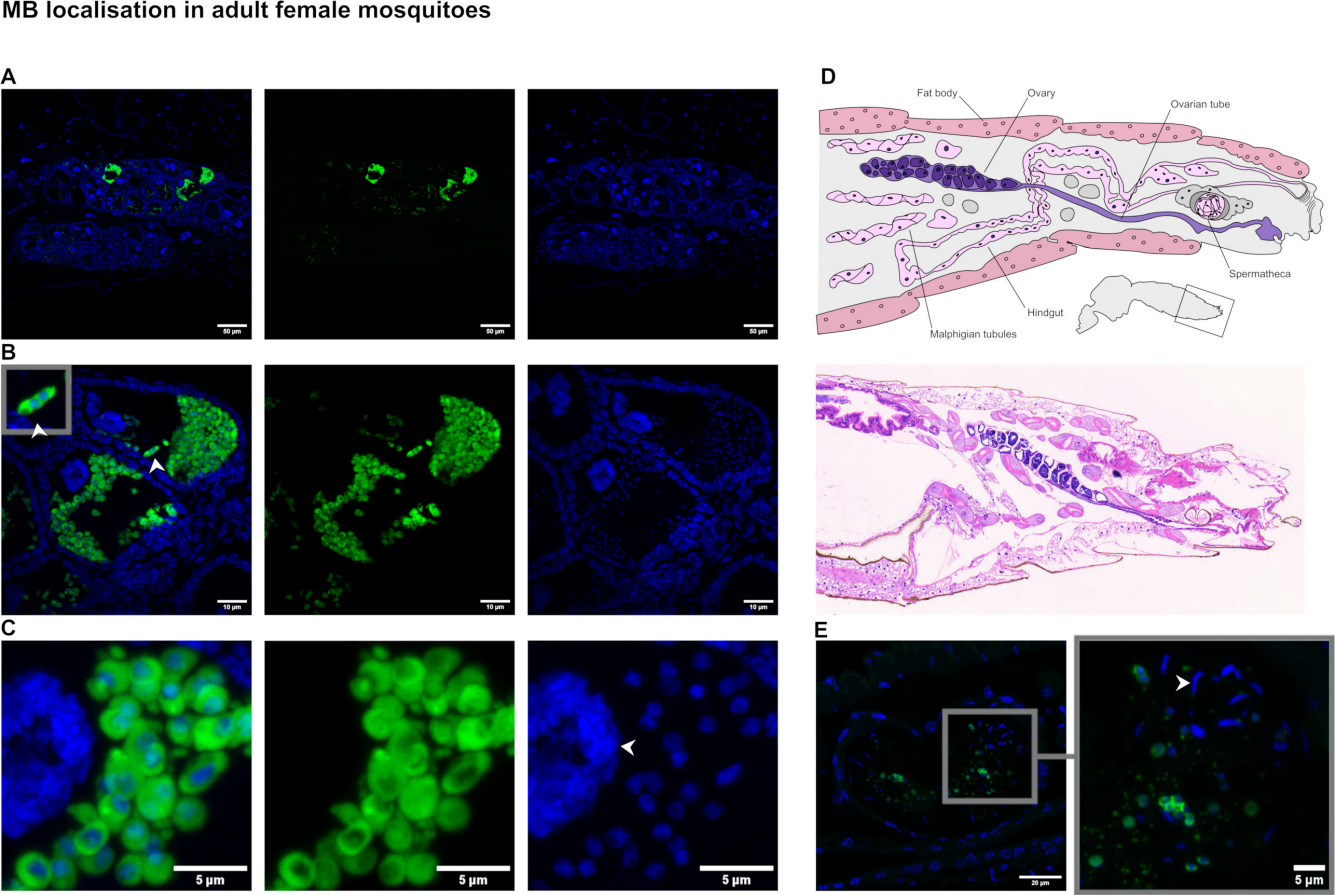
*Microsporidia* sp. MB in adult female ovaries. (**A**) Paraffin-embedded sections of whole female mosquitoes were stained with MB 18S FISH probe (488 green) and Hoechst nuclear stain (blue) to show MB distribution. Images of whole ovaries taken with a 20× objective show variable levels of MB infection between ovarioles. (**B**) A maximum intensity projection of a z-stack taken with a 100× objective lens shows detail of the MB distribution within two ovarioles. A dividing cell is shown enlarged (top left). (**C**) A crop of the same image shows a cluster of MB cells and their nuclei, with a large nurse cell nucleus (labeled) to the left. (**D**) A diagram of the female abdomen showing internal organs and tissues (redrawn using reference 38) compared to a histological section of an MB-positive mosquito stained with H&E. (**E**) Spermathecae from sectioned female mosquitoes (as in panel D) were imaged with a 100× objective as several tiles and stitched together to show MB signal and associated nuclei among the mosquito sperm nuclei (labeled).

FFPE sections of whole adult *An. coluzzii* from a stable MB-positive lab colony were stained, using the same method as above, to determine the tissue localization of MB. In adult females, MB was only found in the ovary with no positive FISH signal found in other tissues (*n* = 16). Midguts were examined in multiple individuals across several experiments but showed no positive MB signal. The quantity of MB in each ovariole of an ovary appears highly variable (Fig. 2A). Within the most infected ovarioles, MB meronts were clustered and densely packed, with some cells containing two nuclei indicating active cell division (Fig. 2B and C). Sectioning of whole mosquitoes (Fig. 2D) occasionally revealed positive MB signal in the spermathecae of mated females (Fig. 2E). Some of these signals were clearly nucleated MB cells as seen in the ovaries, whereas others were much smaller and did not appear to co-localize with any nuclear signal. Observing sperm cells in both the H&E and nuclear stains, with the lack of MB signal in other unfertilized spermathecae, suggests that MB has been transmitted horizontally from infected males.

### *Microsporidia* sp. MB localizes to the testes in adult male *An. coluzzii*

**Fig 3:**
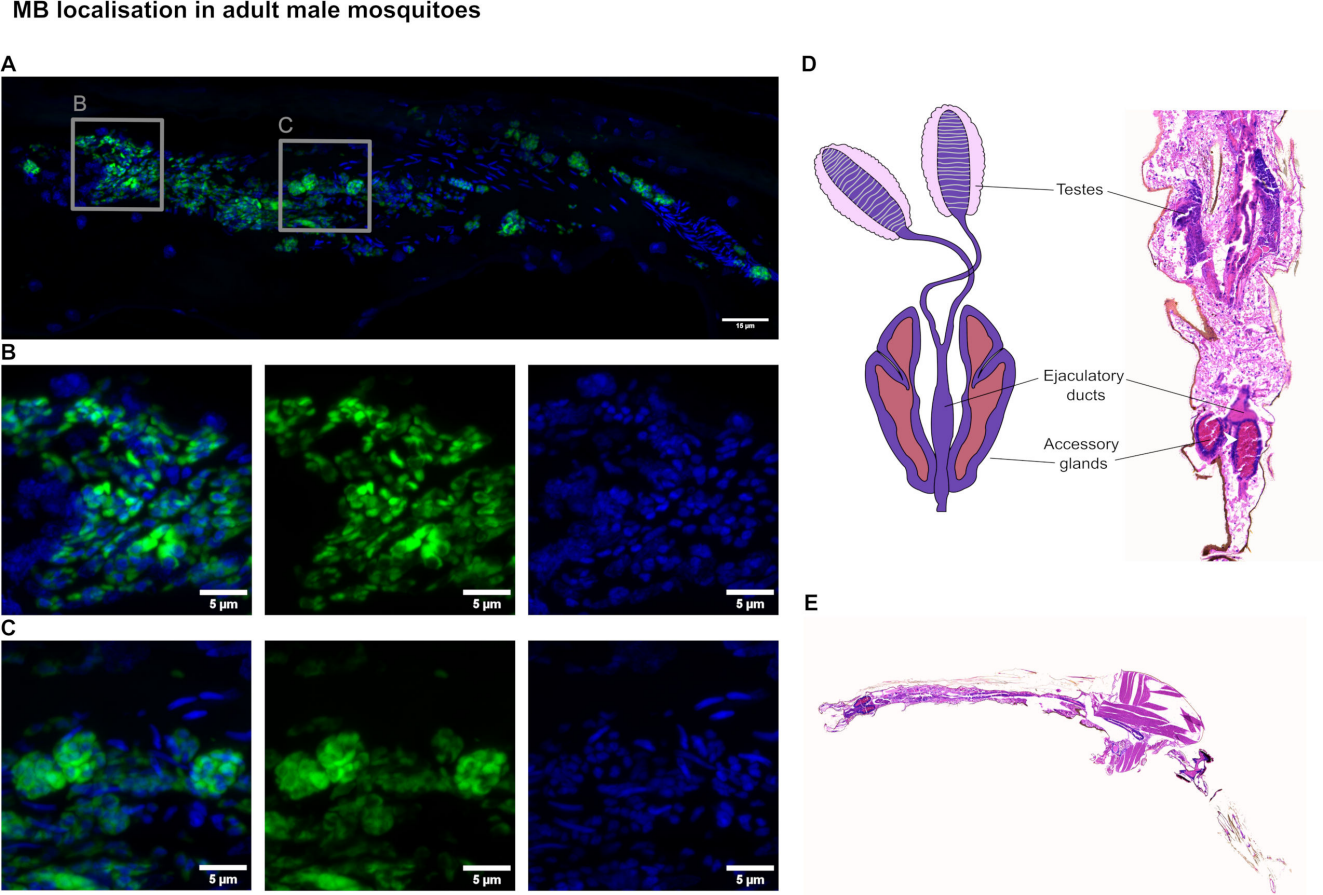
*Microsporidia* sp. MB in adult male testes. (**A**) A tiled image shows the entire testis in high resolution with MB signal (green: Alexa Fluor 488-18S FISH probe) mostly localized to the spermatocytes rather than the fully developed spermatozoa (blue “streaks”: Hoechst). (**B**) At the anterior of the testes, MB can be seen in high abundance with a less clustered distribution. (**C**) More posterior in the testes, *Microsporidia* sp. MB was imaged in spherical clusters (4–6 μm across). All fluorescent images represent maximum intensity projections of z-stacks acquired on a Zeiss LSM710 confocal microscope. (**D**) A diagram of the male gonads and gonoducts (redrawn using references 37, 39) shows the position of the testes in relation to a cropped image of a tissue section of the posterior of an adult male *An. coluzzii*. (**E**) A complete section of an adult male *An. coluzzii* stained in H&E with the accessory glands visible at the posterior.

FISH staining for MB in tissue sections of adult males also revealed gonadal localization. Other than occasional outliers (individual cells), the majority of MB was found in the testes (*n* = 9) (Fig. 3A). Sectioning and imaging of multiple individual males only rarely revealed MB-positive signal in the midgut region, a tissue which was reported as weakly qPCR positive for MB in Makhulu et al*.* (23). The only example of MB signal with clear nuclei found in tissues outside the gonad was a small cluster between the midgut and the cuticle (Fig. S7). No MB signal was seen in other adult male tissues (Fig. S1). In the testes, MB replication appears to be concentrated toward the anterior tip and evenly distributed among the spermatocyte nuclei (Fig. 3B). Toward the posterior, some MB cells were observed in dense spherical clusters of around 4–6 μm in diameter (Fig. 3C). MB were seen centrally in the anterior portion of the testes alongside the majority of mature spermatozoa, but were not detected in large numbers. The locations of the major male reproductive organs are shown in a diagram in Fig. 3D and in a section of a male mosquito, stained with H&E. The forms of MB seen in the male testes are consistent in size and shape with those found in a spermatheca of a mated female in Fig. 2E.

### FISH-stained sections of gravid females reveal octosporogony in developing oocytes

**Fig 4:**
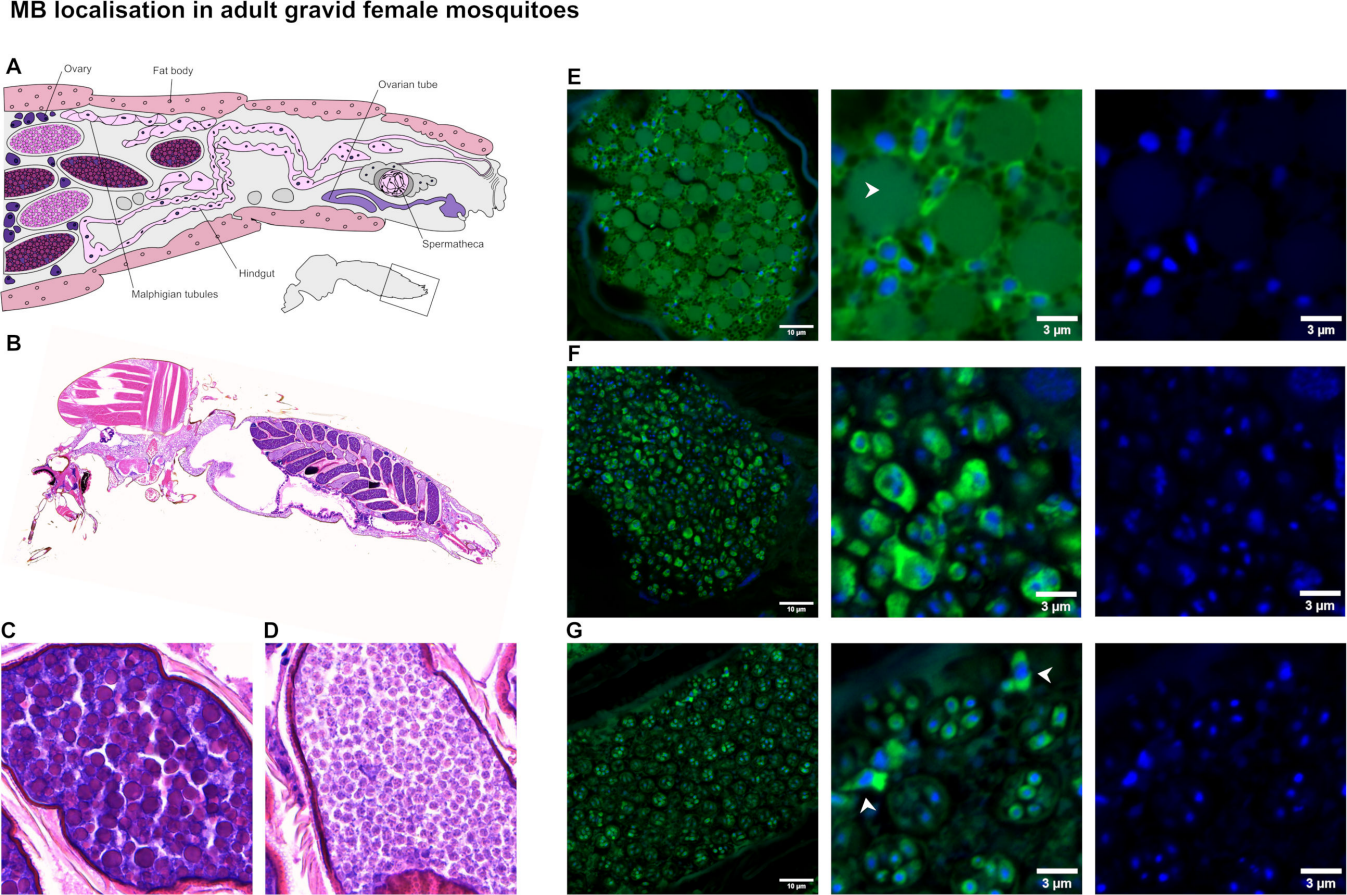
*Microsporidia* sp. MB in gravid female *An. coluzzii*. (**A**) The major features in the posterior abdomen of a gravid female mosquito (redrawn using reference 38). (**B**) An example of FFPE section of gravid female MB-positive *An. coluzzii* 3 days after bloodfeeding stained with H&E. (**C, D**) Two different colors and morphologies can be seen in the developing eggs: “dark” (**C**) and “light” (**D**). (**E**) Alexa Fluor 488-18S FISH probes (green) and Hoechst nuclear stain (blue) were used to stain subsequent sections of the slide from panel A. MB can be seen at the periphery of developing oocytes in “dark” embryos (as in panel C). (**F**) Evidence of dense MB infection in “light” oocytes (as in panel D) presenting as a mix of larger mononucleated cells, large multinucleated aggregates, and clusters of smaller mononucleated cells. (**G**) Most developing oocytes in the “light” form contain only the small mononucleated cells, with each cluster surrounded by a spherical compartment.

Tissue sectioning of gravid females allowed the localization of MB to be investigated in the context of the surrounding mosquito tissues and at the earliest stages of embryogenesis. The locations of the major organs in the gravid female abdomen (Fig. 4A) are shown in a tissue section stained with H&E (Fig. 4B). Stained oocytes appear in two forms: “dark” blue colored (Fig. 4C) and “light” pink colored (Fig. 4D). The “dark” form of the oocyte was the only form present in uninfected gravid females, whereas MB-positive females had oocytes of both forms. In the “dark” form, many yolk granules can clearly be seen (Fig. 4C) inside a single periplasm. In the “light” form, the oocyte was densely packed with round structures, each containing darker puncta (Fig. 4D). Yolk granules were either greatly depleted or entirely absent in “light” form oocytes. The structures seen in H&E-stained sections were investigated with subsequent FFPE sections of whole gravid females (*n* = 9) labeled using MB FISH probes as before. In “dark” form oocytes, brightly labeled elongate MB cells were localized just under the developing chorion (Fig. S3). Oocytes in the “dark” form were also seen containing MB at higher density and dispersed throughout the periplasm, between the yolk granules (Fig. 4E). Some “light” oocytes contained similarly brightly labeled forms, but arranged in multinucleate, spherical clusters (Fig. 4F). The “light” form oocytes mostly contained densely packed clusters of smaller, weakly FISH-labeled MB cells (Fig. 4G). Unlike the brightly labeled clusters in Fig. 4F, these weakly labeled cells were mononucleate, with separate cytoplasmic labeling for each cell and appeared to be within a spherical structure with a diameter of ~4–6 µm.

**Fig 5:**
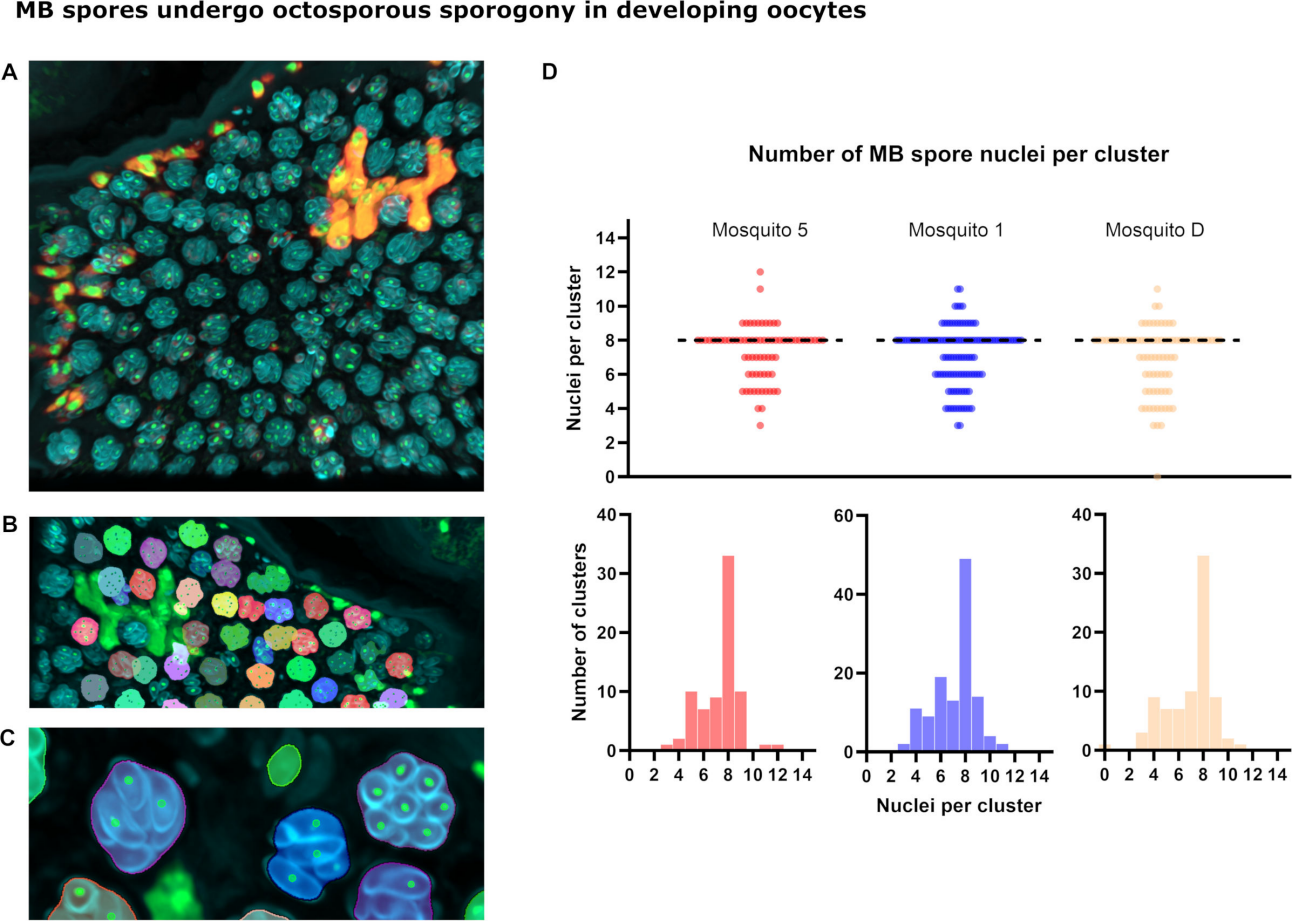
MB spores undergo octosporogony in developing oocytes. (**A**) A three-dimensional shaded volume projection of a z-stack of MB spores within an unfertilized egg, day 3 post-bloodfeeding is shown from a slide stained as follows: red = ATTO 594 MB 18S FISH probes, cyan = Calcofluor White (chitin), green = SYTOX Green (nucleic acid). (**B**) Clusters were segmented using the cyan channel and a series of filters to exclude clusters that intersect the volume boundaries. Nuclei were counted using the green channel. (**C**) A two-dimensional slice of the shaded volume in panels A and B is shown to highlight the boundary of the clusters (various colours) and the counted nuclei (green). (**D**) The segmentation in panels B and C was applied to stacks of two further spore-containing, unfertilized eggs from two other individual mosquitoes stained on the same slide. The resulting data showing the number of nuclei per cluster show a median of eight spores per cluster, suggesting MB undergoes octosporogony at this stage of its lifecycle.

To better visualize the weakly labeled cells in “light” eggs, a new ATTO 594 MB 18S multi-target probe (Table S1) was produced which achieved a brighter signal. Using the new probe on subsequent sections from the same block as above showed some “light” oocytes containing elongate, brightly labeled MB meronts, the intermediate clustered forms, and weakly labeled clusters of smaller MB cells consistent with descriptions of late-stage microsporidian sporogony (Fig. S4). Further sections of gravid females (day 3 post-bloodfeeding) were labeled with the ATTO 594 MB FISH probe, as well as Calcofluor to detect chitinous spore walls and SYTOX Green to detect nucleic acid. The triple stain revealed a bright Calcofluor signal surrounding the small regular cells from “light” phenotype eggs (Fig. 5A). The larger MB cells did not show Calcofluor signal in any stage visualized (Fig. 5A). These data suggest that the “light” phenotype eggs are a site of sporogony due to the presence of positively FISH-stained MB cells with chitinous spore walls. Z-stacks taken at super-resolution from multiple individuals and analyzed in three dimensions showed the median number of nuclei per cluster was eight, indicating MB undergoes octosporogony (Fig. 5B through D).

### Embryo sections show MB localizes during early embryogenesis

**Fig 6:**
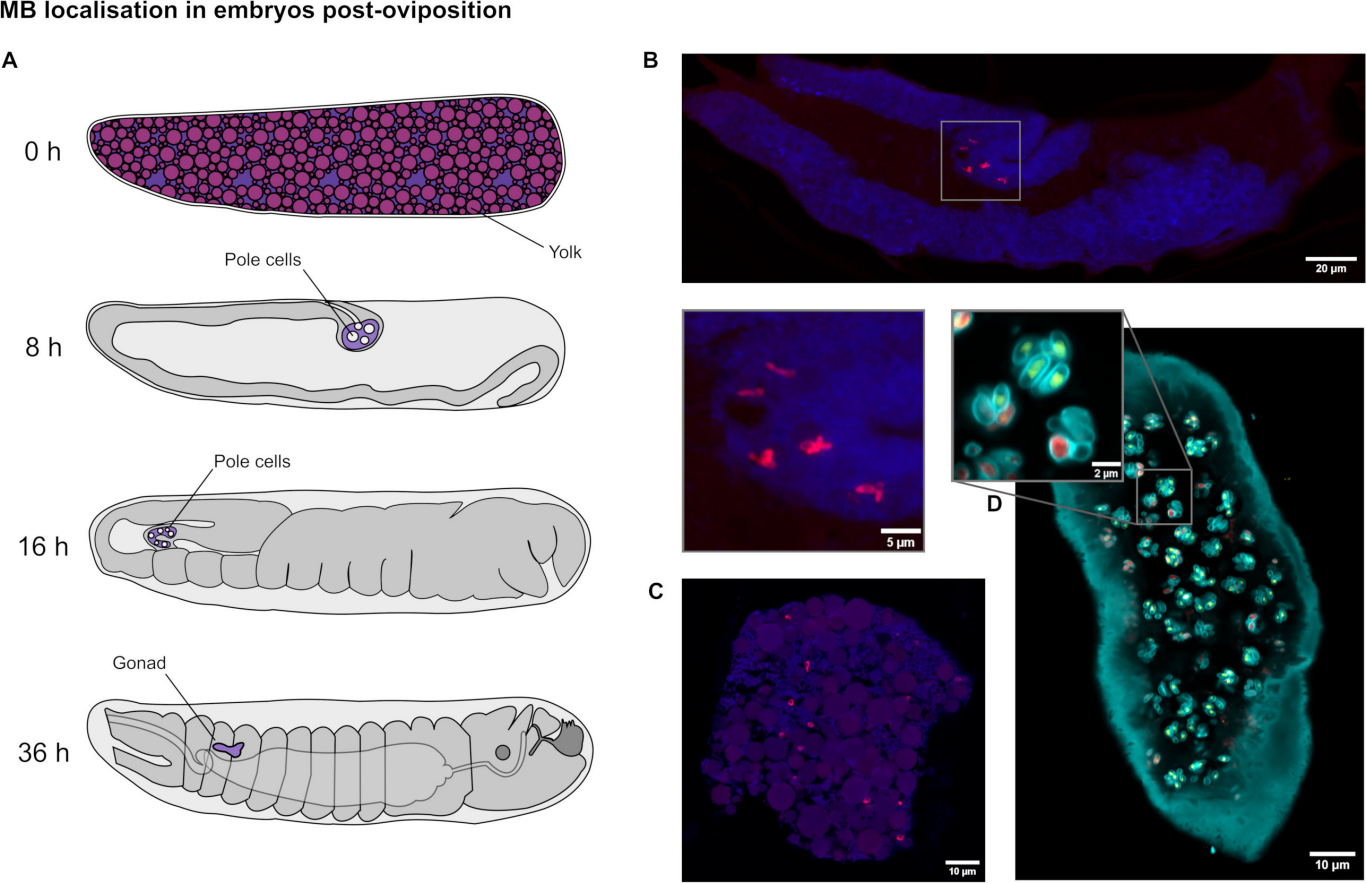
*Microsporidia* sp. MB in embryos post-oviposition. (**A**) Diagrams illustrate laterally the stages of development of mosquito embryos from 0 to 36 h post-oviposition (redrawn using references 37, 40, 41). (**B**) A maximum intensity projection of a tiled z-stack of confocal images showing a section of a developing mosquito egg in the same orientation as the diagrams in panel A. Embryos from this slide had between 0 h and 18 h of development. ATTO 594 MB 18S FISH probes (red) show MB localizing only to a single area, consistent with the location of the pole cells. Nuclear material was stained with SYTOX Green (blue). A magnification of the defined region shows a cluster of MB meronts. (**C**) Further confocal images from the same sections show MB widely distributed in undeveloped eggs, identified by the presence of yolk. (**D**) Some eggs from the same section showed evidence of spores as seen in the gravid females. Lookup tables have been altered as follows to clearly show the spore wall and for comparison to previous images of spores: ATTO 594 MB 18S FISH probes = red, SYTOX Green nuclear stain = green and Calcofluor White = cyan.

Sections of multiple MB-positive embryos, from 0 to 18 h post oviposition, were labeled with the ATTO 594 MB FISH probe, Calcofluor White, and SYTOX Green nuclear stain. The slides showed three different MB localization patterns. Stages of embryo development are described in diagrams (Fig. 6A). Developed embryos, identified by dense host cell nuclei and tissues, had fewer MB cells, confined to a single region of the embryo (*n* = 10) (Fig. 6B). FISH signal at this localized region showed a small number of meronts with similar morphology to those in other stages, sometimes being more fragmented and indistinct. Early embryos, identified by the presence of yolk granules and a lack of nuclei or only peripheral nuclei, had meronts dispersed among the yolk (*n* = 14) (Fig. 6C). Some embryos were seen to contain clustered spores, as seen previously in the gravid females (Fig. 6D), showing that spore-containing eggs survive oviposition (*n* = 4).

### *Microsporidia* sp. MB spores: transmission electron microscopy (TEM)

**Fig 7:**
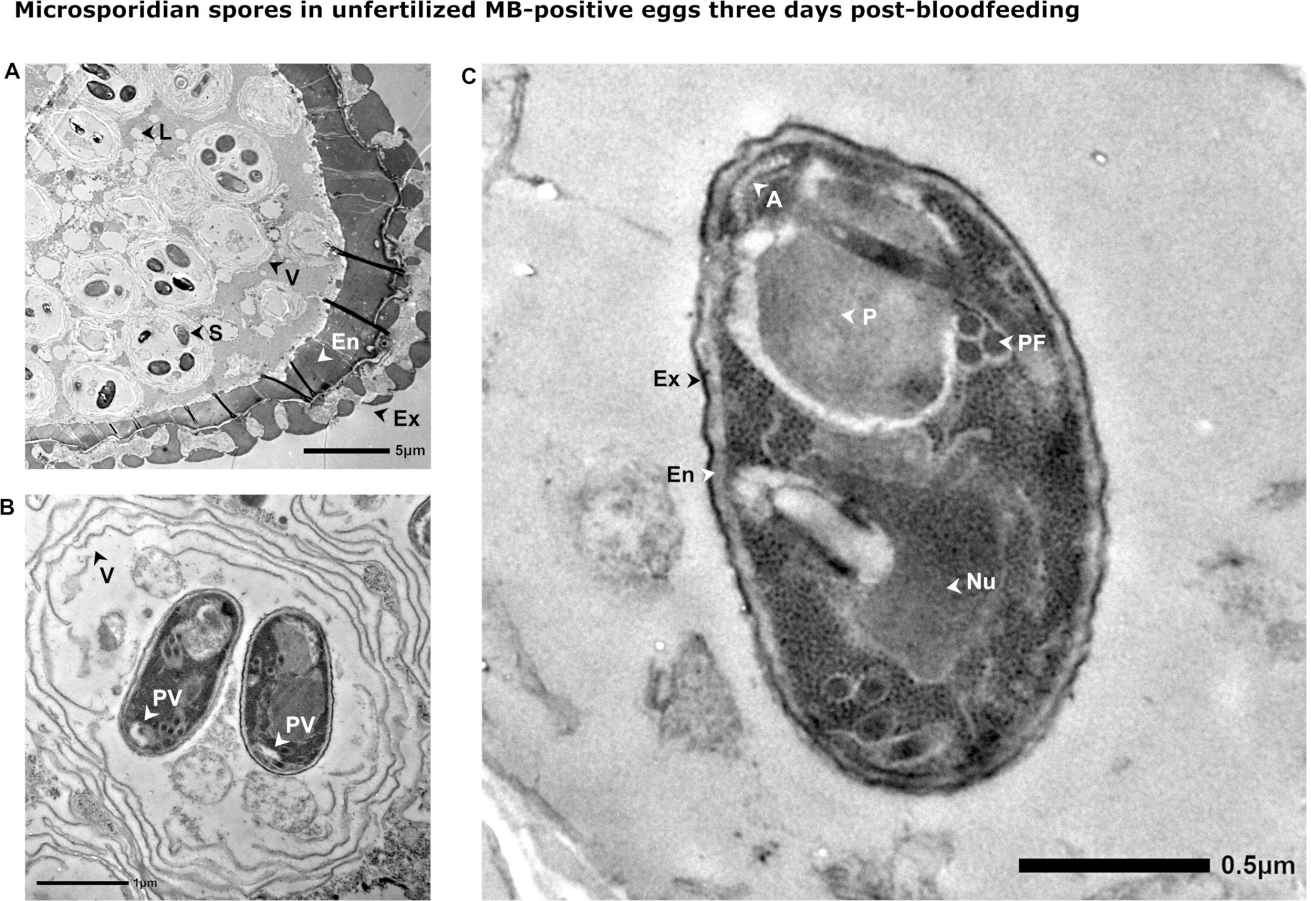
TEM of microsporidian spores in unfertilized MB-positive eggs 3 days post-bloodfeeding. (**A**) A low-resolution image of a section through an unfertilized *An. colluzzii* egg pre-oviposition, showing the layers of the developing chorion on the right, with MB spores visible within vacuolar compartments in the periplasm on the left. En = endochorion, Ex = exochorion, L = yolk lipid, V= vacuole, S = spore. (**B**) A higher resolution image shows two MB spores within a vacuole. The vacuole has several layers of membrane. The posterior vacuole of the spore can also be clearly identified. Pv = posterior vacuole, V = vacuole membrane. (**C**) A section through a single spore shows more characteristic organelles of microsporidian spores. A = anchoring disk, En = endospore, Ex = exospore, Nu = nucleus, P = polaroplast, PF = polar filament.

Higher resolution images of the spores described above were produced using TEM. Low-resolution electron micrographs showed the presence of MB spores in clusters within multiple layers of membrane (Fig. 7A). A parasitophorous vacuole is a common feature in microsporidian sporulation, but the number of layers of membrane observed here is unusual. Further images at higher resolution confirmed MB sporogony, with microsporidian organelles clearly visible including the spore wall, polar filament, anchoring disk, polaroplast, and posterior vacuole (Fig. 7B and C). The length of these spores estimated from optimal transverse sections in TEM images ranged between 1.9 and 2.6 μm (*n* = 14). All spores measured that contained a nucleus appeared monokaryotic, except for an unusually large spore (3.2 µm) that appeared to be malformed. This and another image of a malformed spore are shown in Fig. S5.

## DISCUSSION

Previously published evidence of *Microsporidia* sp. MB blocking *Plasmodium* transmission suggests there is potential to utilize MB as a tool for antimalarial control (20). However, very little is currently known about how MB interacts with its mosquito host, with *Plasmodium*, and the environment in malaria-endemic countries. To develop MB for malaria control strategies, it is crucial to understand its lifecycle, with a particular focus on transmission. Transmission of MB between mosquitoes and its efficiency, whether vertical or horizontal, will be a key factor affecting MB prevalence and dissemination through mosquito populations (24). The promise of naturally occurring malaria-blocking agents is especially salient if they can be transmitted efficiently and maintained at a high level in wild mosquito populations. Eliminating *Plasmodium* transmission without eliminating the vectors would be optimal for minimizing ecological impacts, and methods utilizing natural symbionts are more likely to be straightforward cases for obtaining regulatory approval (42).

There are many species of microsporidia that infect insects, including mosquitoes. Previously described mosquito-infecting microsporidia genera (*Amblyospora, Anncaliia, Culicospora, Culicosporella*, *Duboscqia, Edhazardia, Hyalinocysta, Parathelohania,* and *Vavraia*) (20, 43) are more closely related to each other than they are to MB and tend to be pathogenic to their hosts, although several show delayed pathogenicity in order to allow for vertical transmission. The disadvantage of highly pathogenic vector control agents is that they can be self-limiting, eliminating themselves along with their host. The pathogenicity of these microsporidia often presents as invasion of the fat body tissues or whole adult carcass. In contrast, MB appears to be well tolerated by mosquitoes, with no evidence of destructive adult tissue invasion, and can be stably maintained at high prevalence in lab colonies (D. K. Purusothaman et al., unpublished data; 23). While MB was originally discovered in wild-caught *An. arabiensis* from Kenya (20), all mosquitoes imaged in this paper were from lab colonies derived from *An. coluzzii* from Burkina Faso. MB in both hosts appears similar, but it is important to note the mosquito background and geographic separation of East and West Africa could lead to different phenotypes (25).

Mosquito-infecting microsporidia roughly fall into two groups, (i) those that are more pathogenic to adult mosquitoes, less tissue-specific, and predominantly horizontally transmitted or (ii) those that show delayed pathogenicity in adult mosquitoes, are highly tissue-specific, and rely heavily on vertical transmission (44). The latter group contains species that exhibit variable pathogenicity, adjusting the balance of vertical to horizontal transmission in line with environmental and ecological conditions (45). MB has some similarities to this group, being confined to the ovaries in the female and then sporulating after a blood meal. One difference is that MB clearly replicates within male gonadal tissues and is transferred to the spermathecae after mating (Fig. 2E). Also, there was only limited visual evidence of MB from the lab colony localizing to the midgut or with sections from most individuals lacking MB-positive signal outside the gonads (Fig. S1). This is consistent with previously published imaging data from *An. arabiensis,* where midgut infection was only observed occasionally (25). An MB-positive lab colony from the same Burkina Faso lineage as examined here also strongly inhibits *Plasmodium falciparum* when colony females were challenged with gametocyte-positive patient blood (S. Issiaka et al., unpublished data).

While it is plausible that small, localized areas of MB staining in other tissues may have been overlooked due to the nature of tissue sectioning, the number of sections containing extensive areas of midgut and only positively labeled ovaries suggests that MB does not heavily infect the midgut of *An. coluzzii,* at least under these lab conditions. This suggests that MB localization either changes dramatically under conditions that have not yet been observed, such as in response to *Plasmodium* infection, or that the blocking effect is indirect. Possible indirect effects could include the release of antimalarial compounds, priming of the host immune system, and utilization of resources such as lipids or essential amino acids with a host-wide depletion effect. It is interesting that, similar to fat body infections with other microsporidia, the oocytes containing MB have large quantities of lipid (yolk granules). The yolk appears to be entirely depleted and replaced with spores 2 or 3 days after bloodfeeding in spore-producing oocytes, leading to the “light” phenotype. The depletion is likely due to MB using the host lipid to fuel sporogony, as decreases in lipids, sugars, and glycogen have been seen in other microsporidian parasites of mosquitoes (46). In the “dark” phenotype, the yolk appears to persist until after oviposition when the tissues of the developing embryo form, as in MB-negative embryos.

Fluorescence and electron microscopy revealed octosporogony in MB, a phenotype commonly observed in other microsporidian genera (47–55). A typical example is *Amblyospora connecticus,* which infects *Aedes cantator*, producing a multinucleate sporogonial plasmodium in the fat body that divides into eight sporoblasts. These sporoblasts mature into meiospores enclosed in a sporophorous vacuole (52). Electron microscopy of MB also shows a sporophorous vacuole that appears to either contain multiple bilayers or else is highly invaginated (Fig. 7). While some mosquito-infecting microsporidia (*Edhazardia aedis, Hyalinocysta expilatoria,* and *Vairimorpha* sp.) show abortive sporogony, comparison of electron microscopy images showed that this is unlikely to be the case in MB. Fluorescence microscopy of MB spores showed weaker MB 18S FISH signal than in meronts when using either 18S probe. This is consistent with rRNA and active transcription being lower during sporulation, providing fewer probe binding sites (56). This can be seen clearly when meronts and spores, within oocytes, are imaged together in a field of view under the same laser settings (Fig. S4).

The most closely related microsporidia to MB (based on ribosomal RNA region [MT160806.1]) are the uncharacterized isolates from Colombia, identified in *Culex nigripalpus* and in the sand fly *Pintomyia pia* (57). Also closely related and uncharacterized are Microsporidium PL03 identified in *Culex* sp. and NR-2013, identified in the crustacean genus *Artemia* (58, 59). The closest microsporidian to MB with a characterized lifecycle is *Crispospora chironomi,* found in the non-biting midge (Chironomidae) *Chironomus plumosus* (60). Unlike MB, there is no evidence of octosporogony in *C. chironomi*, which infects the midgut and produces both diplokaryotic and monokaryotic spores in the same cells. While most meronts and all spores observed appear monokaryotic in MB, evidence of binucleate meronts was found in the ovaries of the adult females (Fig. S6).

It is interesting to note that the majority of microsporidia alongside MB in the family Mrazekiidae are hosted by either insects or crustaceans (59). While there was no evidence of alternative MB hosts thus far, several other mosquito-infecting microsporidia have a secondary crustacean host as part of a digenetic lifecycle, so this possibility should not be ruled out (24, 43, 47, 52, 53, 55, 61). Comparisons between MB and other characterized mosquito-infecting microsporidia are interesting in the context of understanding patterns in transmission and adaptation to this niche. However, taxonomic separation between MB and this distant clade means predictions of similar traits and adaptations should be made cautiously.

**Fig 8:**
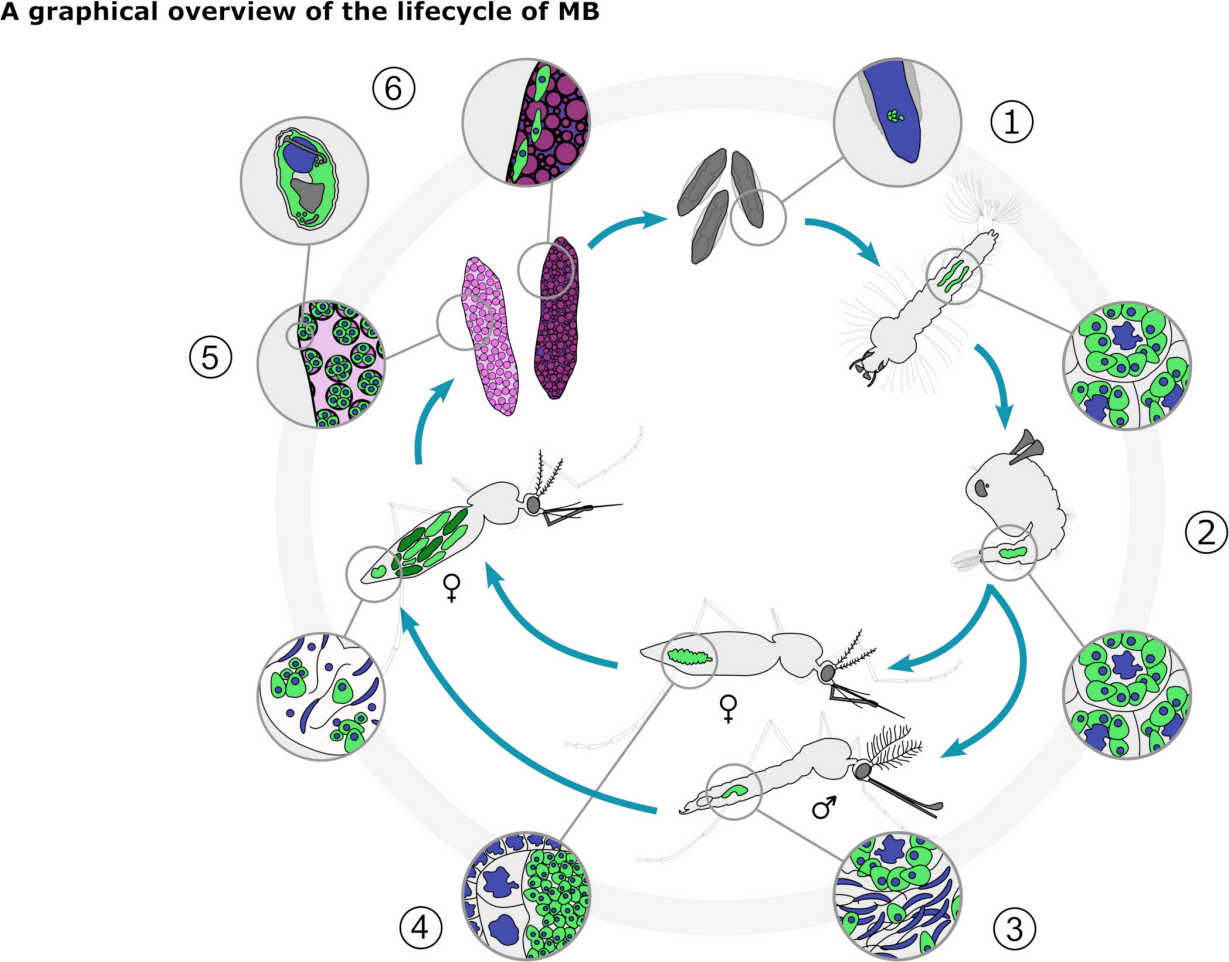
A graphical overview of the lifecycle of MB in a lab colony of *Anopheles coluzzii* from western Burkina Faso. MB localized to the developing gonad in embryos post-oviposition (1), where it persists in developing gonad tissues during the larval and pupal stages (2). In adult male *An. coluzzii,* MB localizes to the testes (3) and can be passed during mating to the female spermatheca. In adult female *An. coluzzii*, MB replicating in the ovaries (4) either persists in an oocyte as meronts or sporulates, densely populating the developing egg with spores. Post-oviposition, the eggs containing MB spores are fragile and likely burst in the water, releasing spores to the environment (5). The eggs only containing meronts develop, with MB localizing to the gonad to continue vertical transmission (6).

Finding sporogony in MB reveals several important points that require further investigation. It is likely that spores containing eggs are laid on the surface of larval breeding sites because they were seen in sections of eggs post-oviposition. It is crucial to investigate whether these spores infect mosquito larvae that would be present in these sites. A graphical overview of the lifecycle of MB as it is currently understood, including the sporulating stage, is shown in Fig. 8. Full characterization of the lab colony and spore challenge experiments on larvae is underway (Purusothaman et al., unpublished). This could be an important alternative or additional method of MB dispersal in the field. Spores could be easier to produce than MB-positive females, especially if they were introduced to cell lines and could be grown at scale. This would greatly improve the scope and scale of large cage trials and semi-field experiments. If the MB spores described here do not infect mosquitoes, they may infect secondary hosts in the larval breeding sites. Three crustacean genera, *Mesocyclops, Macrocyclops,* and *Daphnia,* were screened by Nattoh et al. (24), showing no evidence of MB infection in two field locations. However, sample numbers were low, and MB secondary hosts have not yet been investigated further. As is the case in many other insect-infecting microsporidia (44), there may also be additional MB spore stages in the laboratory or field lifecycle that have not yet been identified. Further work will utilize the characteristics of MB spores along with genomic data to confirm the phylogenetic relationships between MB in West Africa, East Africa, and other microsporidia. MB phylogenetics and genomics can be used to better design *Plasmodium*-blocking experiments, considering the effects of different MB populations as well as the host background when identifying the best candidates for malaria control strategies.

## MATERIALS AND METHODS

### Mosquito rearing

MB-positive and -negative *An. coluzzii* colonies from western Burkina Faso were established at the University of Glasgow (full details of colony establishment and characterization in preparation [Purusothaman et al., unpublished]). Rearing took place at 28°C and 80% humidity on a 12 h day-night cycle. Liver powder (Now Foods) was fed to early instars, then Tetra TabiMin (Tetramin) tablets from L2 onward. Fourth instar larvae were taken and fixed for imaging at days 8–9 post hatching. Adult mosquitoes were maintained *ad libitum* on a 5% sucrose solution. Adult females were fed using a membrane feeding system (Hemotek) and defibrinated horse blood (E&O Laboratories). Embryos were collected at day 1 as per the MR4 manual (62) 24 h post hatching (first instar or L1), 2 days post hatching (second instar or L2), 5 days post hatching (third instar or L3), and 6 days post hatching (fourth instar or L4).

### Preparing samples for formaldehyde-fixed, paraffin-embedded sections

Adult mosquitoes were prepared for sectioning by removing legs and wings before fixing in 4% paraformaldehyde with 0.01% Triton X-100 for 2 h (63). Making a single ventral puncture in the abdomen close to the thorax aided fixative perfusion. Following phosphate-buffered saline (PBS) washes, a nitrocellulose membrane disk (Sartorius, Germany) was loaded with enough carnauba wax to cover both sides while being heated to 75°C on a flat piece of aluminium foil with a digital hotplate. Mosquitoes (*n* = 10–30) were dried of excess PBS and mounted on the disk. The foil and disk were removed from the hotplate and cooled rapidly, securing mounted samples. Wax disks with samples were processed on an Excelsior AS (Epredia), placing the embedding cassette into 70% ethanol at 30°C, then processed through the following cycles: 100% ethanol 30, 40, 40, 45, 45, and 60 min at 35°C, xylene 30, 30, and 30 min at 35°C, and wax 45, 45, and 60 min at 62°C under vacuum. Embedding was carried out on a Histostar (Thermo Scientific), and 3 µm sections were produced with a microtome. Some sections were stained with H&E to check tissue integrity. Histology processing and H&E staining were carried out by the Histology Research Services Laboratory, School of Biodiversity, One Health & Veterinary Medicine, University of Glasgow.

### FISH probe production

A FISH probe targeting the 18S ribosomal RNA region (eukaryotic small ribosomal subunit: NCBI:MT160806.1) from *Microsporidia* sp. MB was produced as in Herren et al. (20), but with a 488 Alexa Fluor fluorophore (5′-Alexa Fluor488-CCCTGTCCACTATACCTAATGAACAT-3′, Integrated DNA Technologies, Belgium). A secondary 18S FISH probe was produced in-house using previously published protocols (64–66). In brief, oligos were designed against the MT160806.1 region with Stellaris probe designer and BLAST, giving 22 different probe sequences that bind specifically to the MB genome. Oligos of 18–22 nt with at least 3 nt spacing were ordered from IDT at 200 µM concentration in Integrated DNA Technologies EDTA (IDTE) buffer and pooled (Table S1).

ATTO 594 N-hydroxysuccinimide-ester (NHS-ester) (ATTO-TEC GmbH) was reconstituted to 20 mM in dimethyl sulfoxide (DMSO), and amino-11-ddUTP was reconstituted to 10 mM in 0.1 M NaHCO_3_ (pH 8.3). A 2:1 molar ratio of ATTO 594 NHS-ester to amino-11-ddUTP was mixed for 3 h in the dark at room temperature, gently shaking. Also, 1 M Tris-HCl (pH 7.4) was added to a final concentration of 10 mM to quench unreacted NHS-ester groups. Concentration of dye-ddUTP mix was adjusted to 1 mM with nuclease-free water.

A terminal deoxynucleotidyl transferase (TdT) reaction mix (15 µL) was prepared as follows: 5 µL pooled oligos (200 µM), 4 µL dye-ddUTP (1 mM) mix, 3 µL TdT buffer (5×), 0.6 µL TdT enzyme (Thermo Scientific), and 2.4 µL nuclease-free H_2_O. The mix was incubated at 37°C overnight and purified using the Zymo Oligo Clean & Concentrator Kit (Zymo Research) protocol, initially adding 100 µL oligo binding buffer. Probe concentration was measured by nanodrop and adjusted to 25 µM.

### FISH staining protocol

Paraffin wax was removed from sections using Histo-Clear for 5 min, followed by a graded ethanol series: 100%, 100%, 70% of 30 s each followed by H_2_O. As an optional step to increase DNA accessibility: slides were immersed in 2× saline-sodium citrate buffer (0.03M sodium citrate [pH 7.0], 0.3M NaCl) and boiled at 75°C in a water bath for 20 min. Slides were washed in H_2_O, and peroxidase-antiperoxidase (PAP) pen (Daido Sangyo) was used to outline the sample. Hybridization buffer was prepared as described in Ant et al. (67). All water was removed, and enough hybridization buffer was added to cover each sample. The slides were placed in a sealed humidity chamber and protected from light for incubation at 37°C for 12 h. Hybridization buffer was removed, and slides were washed in water three times. Slides were dried thoroughly and mounted in ProLong Glass Antifade Mountant (Thermo Fisher Scientific) with a coverslip.

Stains were applied successively or individually depending on the sample, before a final wash in water: Calcofluor White at 1 mM with 0.4 mM Evans Blue Dye (Bactidrop, Thermo Fisher Scientific) for 10 min, SYTOX Green (Thermo Fisher Scientific) at 1 µM for 10 min, and Hoechst at two drops/mL (NucBlue, Thermo Fisher Scientific).

### Light microscopy and image processing

For confocal microscopy, a Zeiss LSM980 with Airyscan, a Zeiss LSM880 with Airyscan, and a Zeiss LSM710 were used with settings standardized between positive and control samples. All channels were detected separately with unique detector wavelength ranges. Images were processed using ImageJ FIJI and the QuickFigures plugin (68). Image brightness and contrast were adjusted linearly for each image to display MB clearly. Large differences in MB signal and tissue background signal between stages make this approach necessary (Fig. S4). For this reason, comparisons of intensity between images are not meaningful. Statements on intensity derive from comparisons between objects within a single field of view. When describing the number of individuals imaged with a certain phenotype, *n* = 4 means four different individuals imaged with the phenotype described. Multiple sections of a mosquito may have been imaged, with some sections containing a phenotype and some not, but are counted as *n* = 1. Image replicates of different lifecycle stages and phenotypes are listed in Table S2. Diagrams were drawn in Inkscape v.1.4 using licensed reference sources (36, 38–40).

### Transmission electron microscopy

Mosquito abdomens were fixed in 2.5% glutaraldehyde and 4% paraformaldehyde in 0.1 M cacodylate buffer (pH 7.2), washed in 0.1 M cacodylate buffer (pH 7.2), and post-fixed in 1% OsO_4_ for 1 h on ice. After several washes in 0.1 M cacodylate buffer, the samples were stained all together with 0.5% uranyl acetate in water for 30 min. Samples were washed with water, dehydrated in an ascending acetone series (30, 50, 70, and 100%), and epoxy resin embedded. Ultrathin sections were collected and imaged on a JEOL 1400 transmission electron microscope (JEOL) operating at 80 kV.

### Measuring MB spores

TEM images of gravid female tissue sections, 3 days post-bloodfeeding, were used to estimate the size of MB spores. Typical transverse spore sections were selected as a subset of the data and were measured in FIJI (69).

## SUPPLEMENTAL MATERIAL

Supplemental material msphere.00851-24-s0001.docx Figures S1-S7; Tables S1 and S2.

ASM does not own the copyrights to Supplemental Material that may be linked to, or accessed through, an article. The authors have granted ASM a non-exclusive, world-wide license to publish the Supplemental Material files. Please contact the corresponding author directly for reuse.
