# Supplementary material for: Imaging the lifecycle of Microsporidia sp. MB in Anopheles coluzzii from western Burkina Faso reveals octosporogony
Source: mSphere. 2025 May 22;10(6):e00851-24. doi: 10.1128/msphere.00851-24 (PMC12188704; doi:10.1128/msphere.00851-24)
Supplement: Supplemental material — Figures S1-S7; Tables S1 and S2. [file msphere.00851-24-s0001.docx]

Imaging the lifecycle of Microsporidia sp. MB in *Anopheles coluzzii* from western Burkina Faso reveals octosporogony:

**Supplementary Figures**


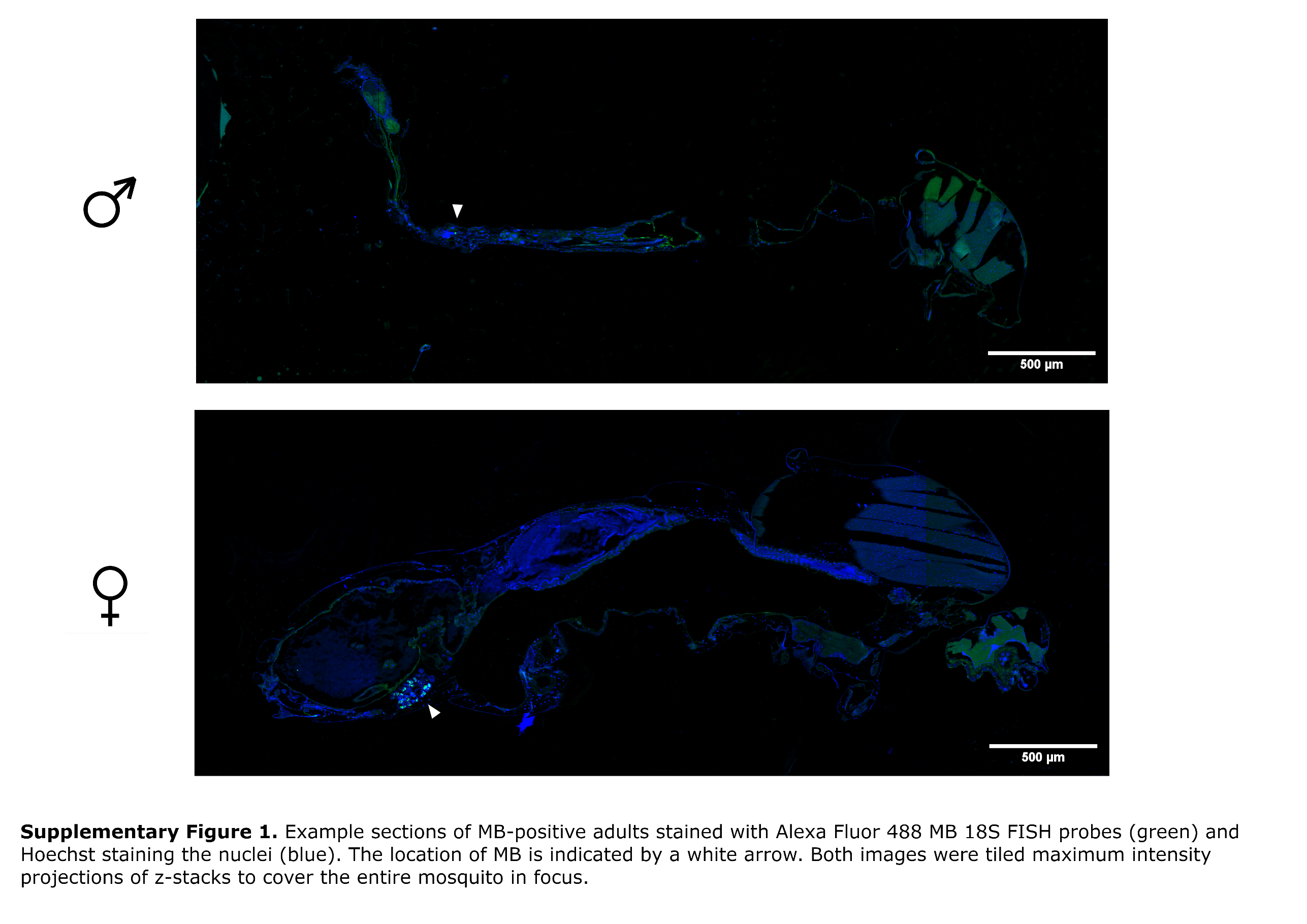


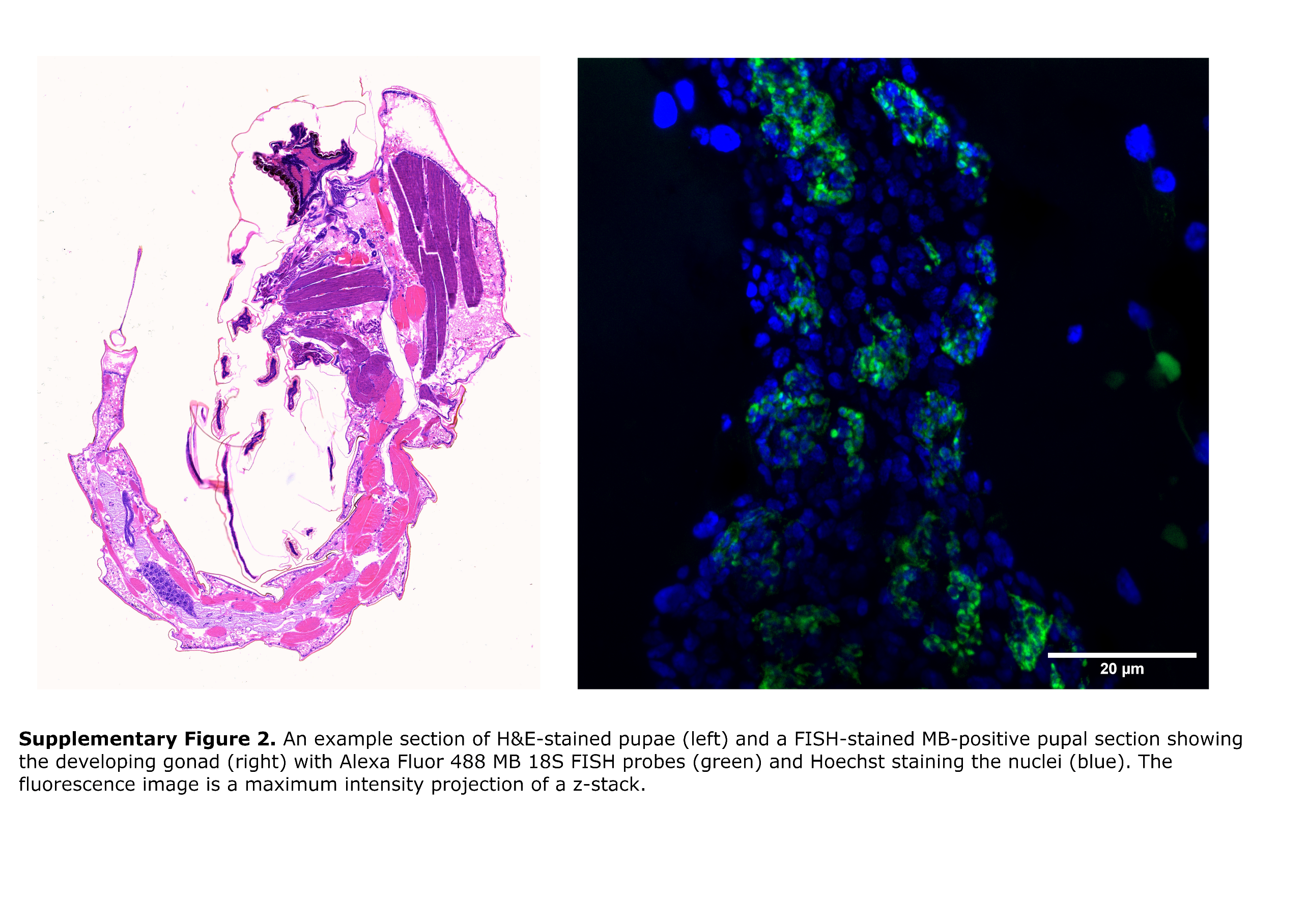


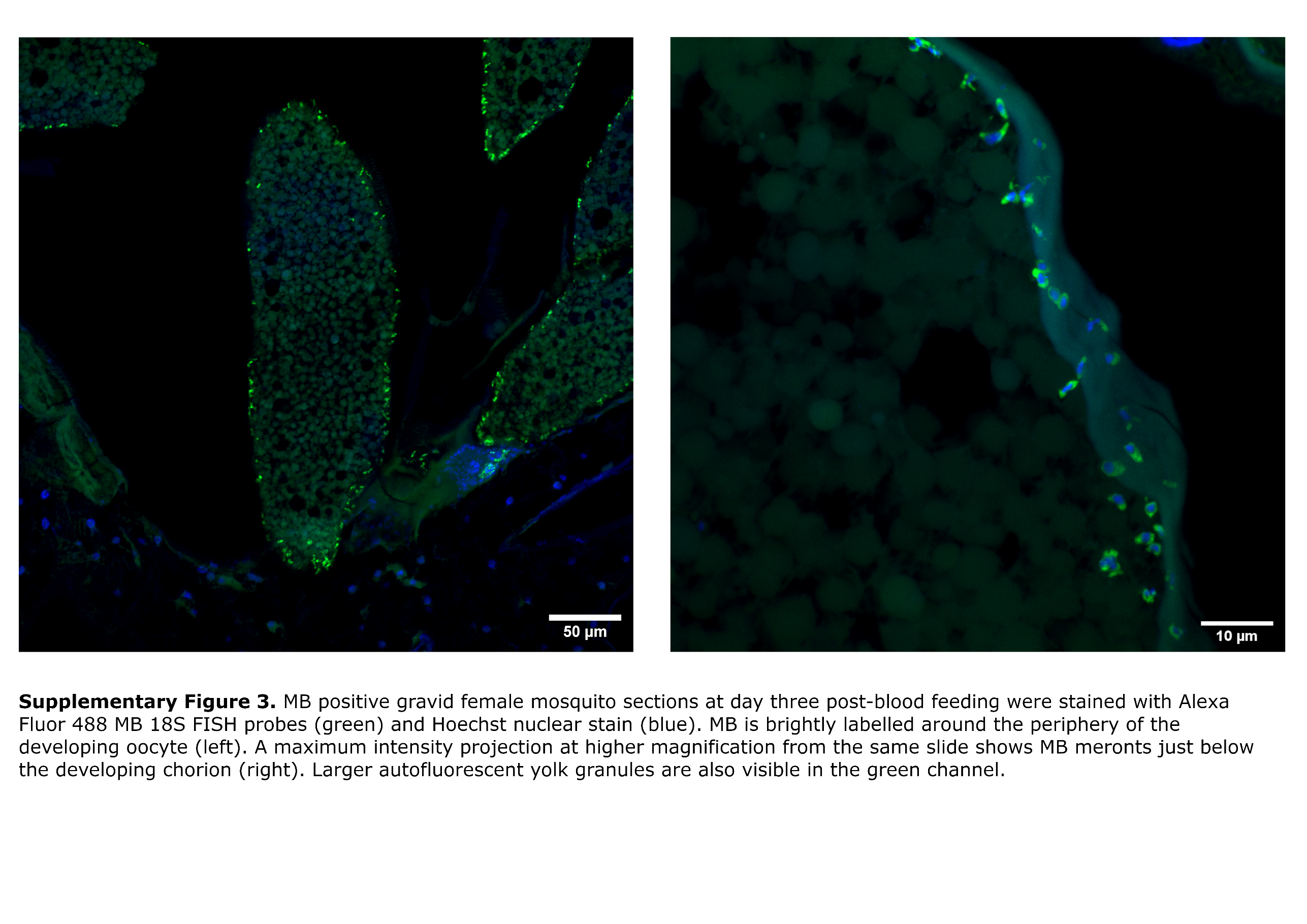


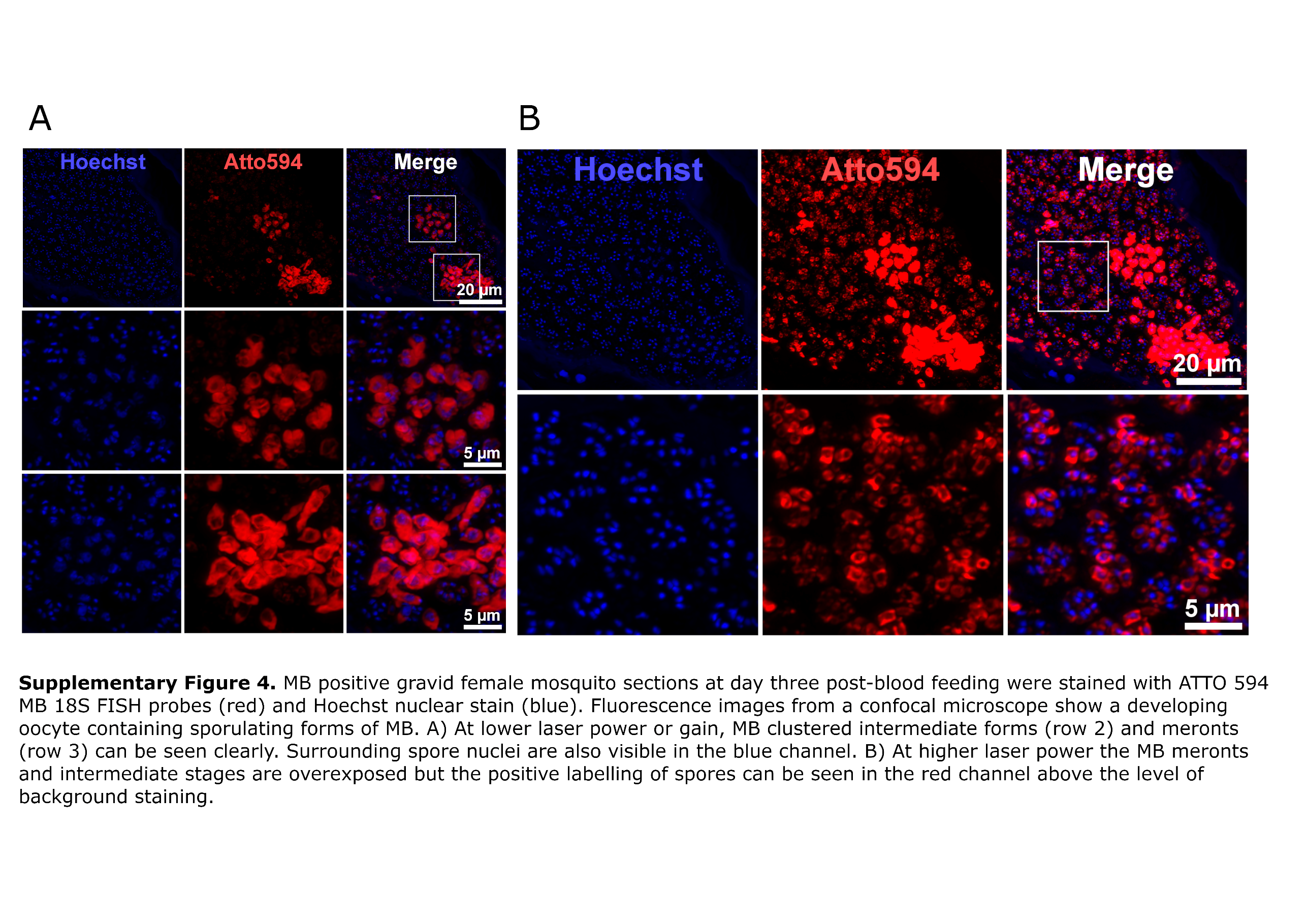


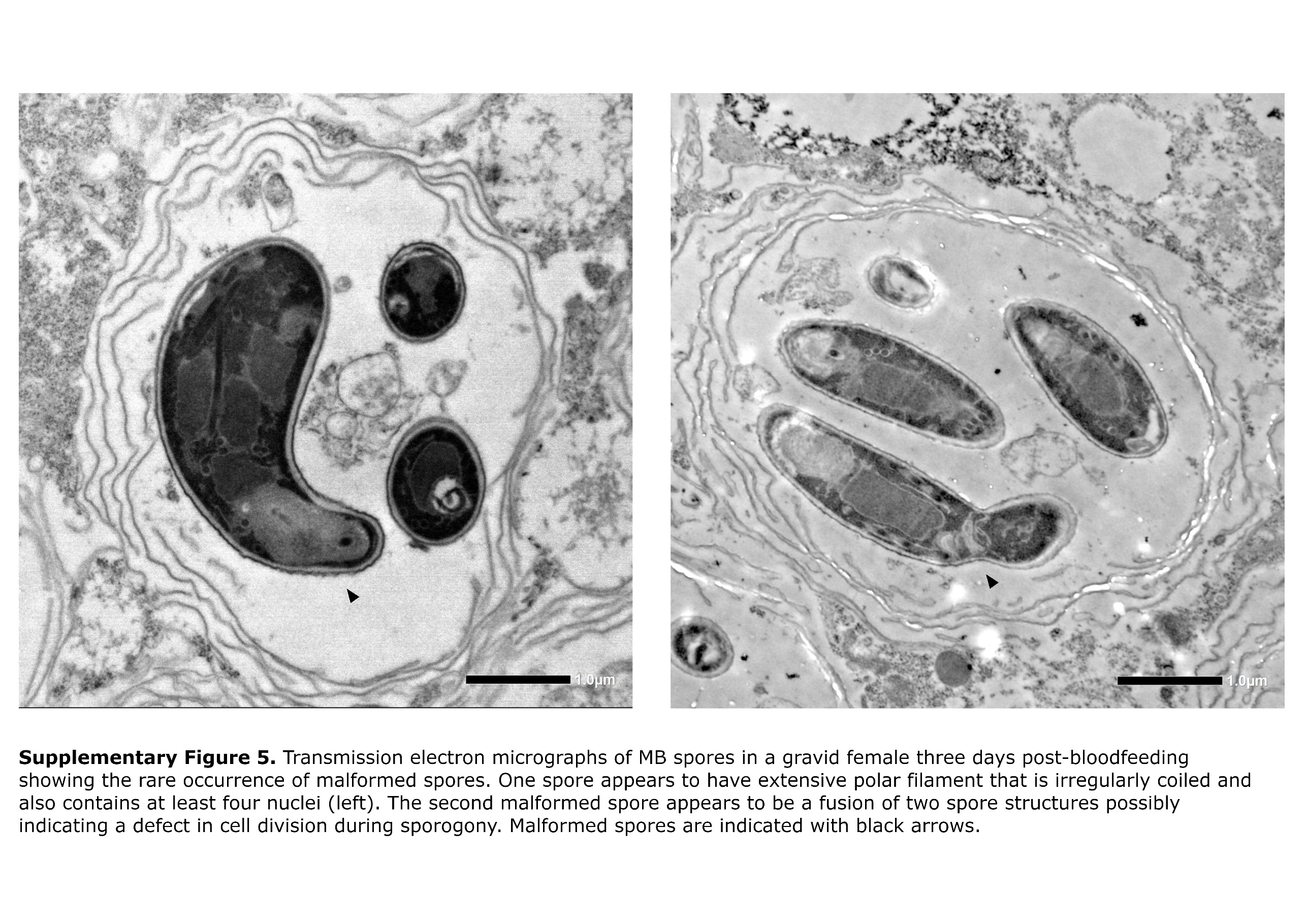


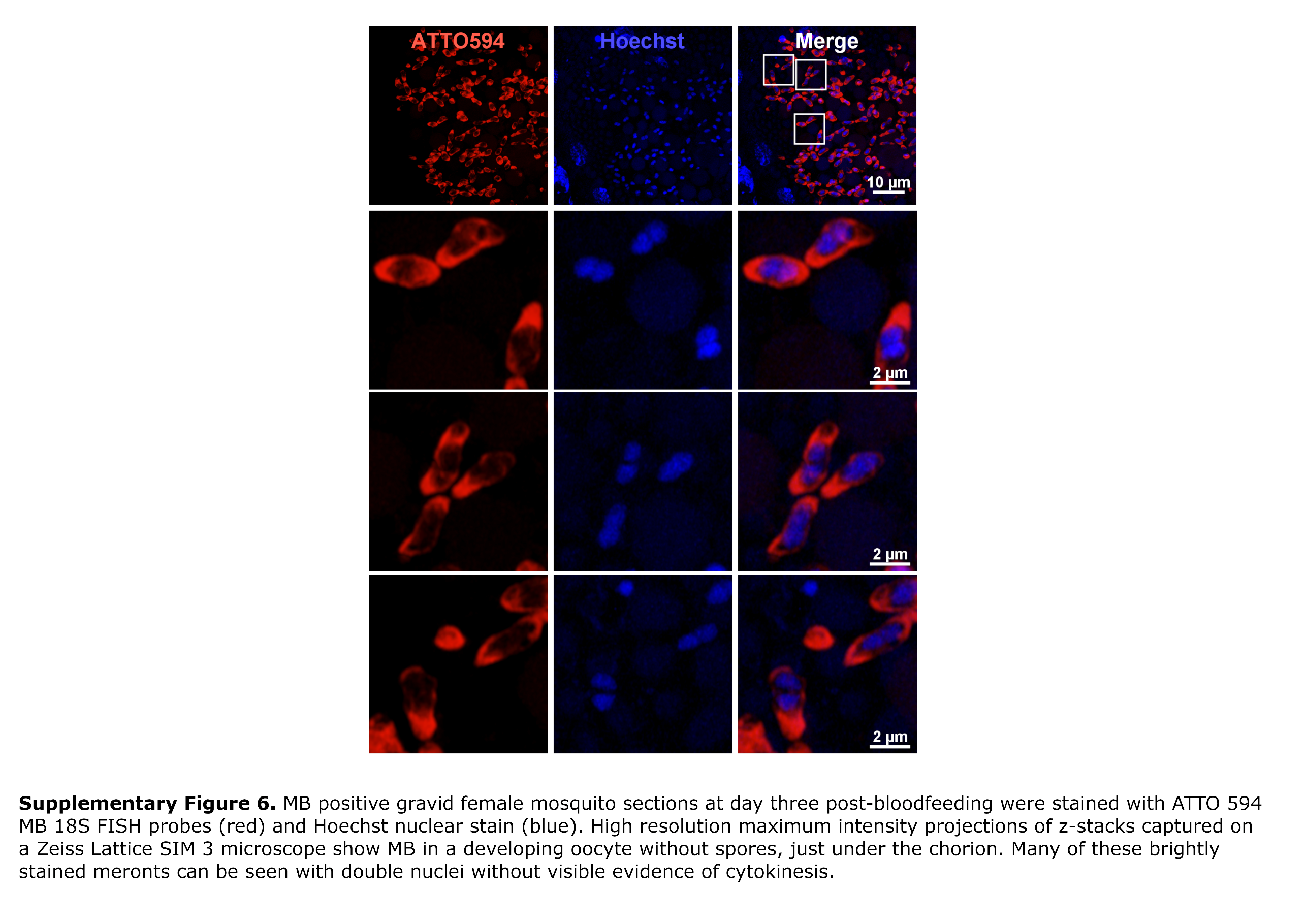


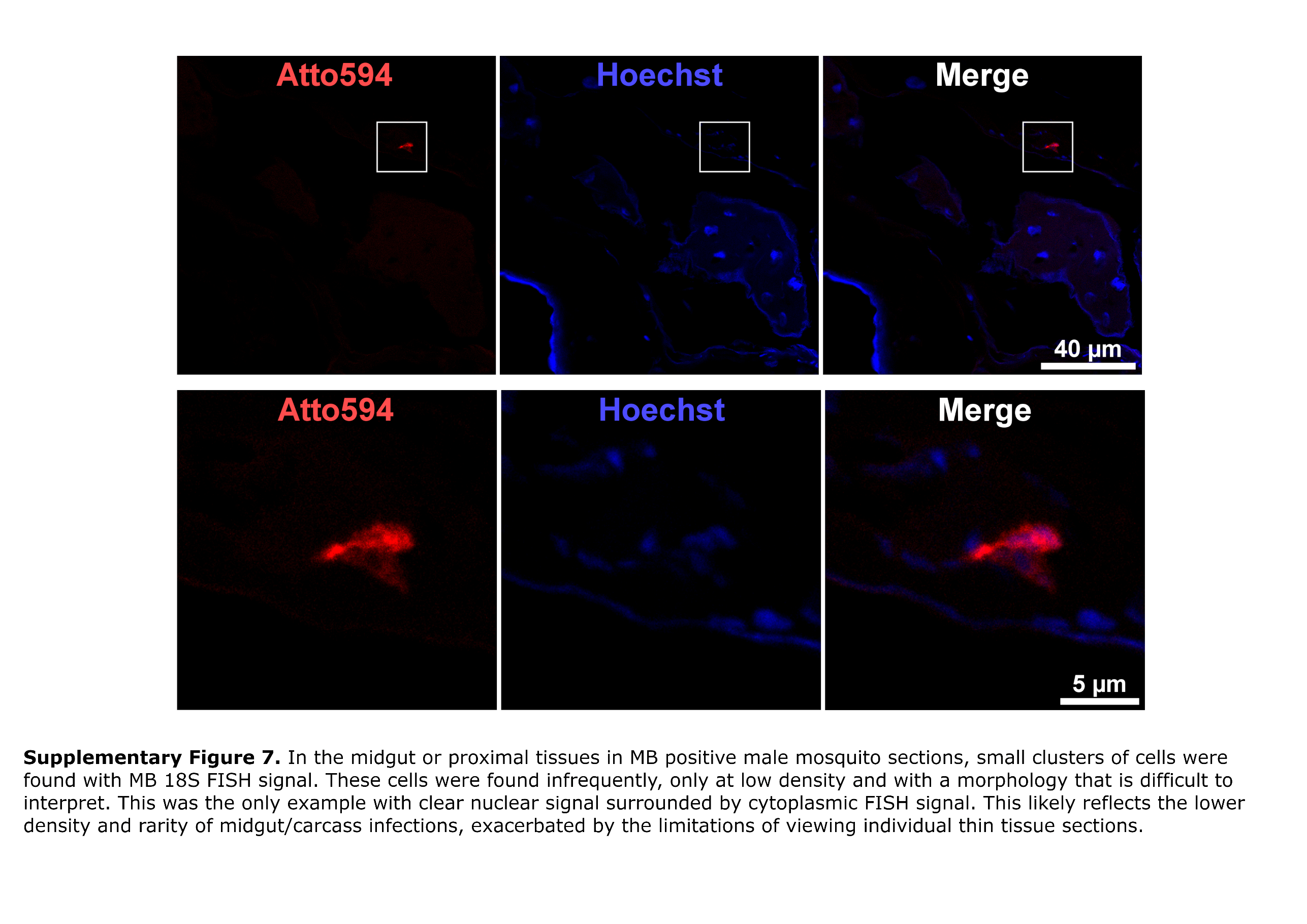

**Supplementary table 1.** Sequences of 22 probes that bind the 18S ribosomal region of Microsporidia sp. MB (NCBI:MT160806.1). These probes were ordered and mixed, before labelling with ATTO 594 and subsequent purification (methods).

**Supplementary table 2.** A summary of all individual mosquito sections imaged and number of independent experiments in which they were observed. In each case the number reflects the total where the phenotype presented was observed and imaged, not the total number of mosquitoes observed or imaged.
